# Experimental study on the homogeneity of microbial grouting to reinforce calcareous sand

**DOI:** 10.1371/journal.pone.0335401

**Published:** 2025-12-03

**Authors:** Xiaoyan Ding, Ping Li, Xiaoyong Mao, Xinlei Zhang

**Affiliations:** 1 Institute of Intelligent Manufacturing and Smart Transportation, Suzhou City University, Suzhou, China; 2 College of Urban and Rural Development, Fuyang Institute of Technology, Fuyang, China; 3 Research Center of Urban Underground Space, Nanjing Tech University, Nanjing, China; China University of Mining and Technology, CHINA

## Abstract

Microbial-induced calcium carbonate precipitation (MICP) technology has shown remarkable potential in environmental engineering fields, such as soil improvement, pollution control and hydrological barrier construction. However, when applied to the reinforcement of sandy materials, the main challenge of MICP technology is the nonuniformity of the curing effect, which greatly restricts the application of this technology in practical engineering. The aim of this study is to explore and optimise the application of MICP technology in calcareous sand reinforcement to improve the uniformity and efficiency of the reinforcement. By treating calcareous sand samples from an island in the South China Sea with Pasteurella spp. octococcus and conducting 75 unconfined compressive tests, the effects of different filling rates (0.5, 3, and 7 mL/min), cementing solution concentrations (0.25, 0.5, 1, and 3 mol/L), and numbers of filling rounds (2, 4, 6, and 8 rounds) on the homogeneity of the reinforcement were systematically investigated. The best curing effect was achieved at an infusion rate of 3 mL/min, which improved the strength of the soil while maintaining a high degree of uniformity. A good balance between compressive strength and uniformity was achieved at cement concentrations ranging from 0.5 mol/L to 1 mol/L. Increased grouting effectively improved the distribution uniformity of the MICP cemented structure. The destructive strain εf of the cured specimens ranged from 1.5% to 6%, which was inversely proportional to the peak strength qu; the elastic modulus E50 was positively correlated with qu. The stress‒strain curves were characterised by three phases of slow increase, rapid increase and sudden decrease in stress under different binder concentration conditions. For the unevenly cured samples, the stress‒strain curves were disordered, and there were multiple stages of stress peaks. This work confirmed that the MICP technique can significantly enhance the mechanical properties of calcareous sand, but precise control of the filling parameters is needed to ensure the uniformity and efficiency of the reinforcement. The suggested optimised parameters are as follows: a filling rate of 3 mL/min, a cementitious solution concentration of 0.5 mol/L to 1 mol/L, and a reasonable number of filling. This study provides a theoretical basis and practical guidance for the application of MICP technology in calcareous sand reinforcement scenarios.

## 1. Introduction

Soil improvement plays a pivotal role in ensuring the stability and durability of geotechnical structures, particularly in marine and coastal areas where foundation soils are typically loose, weak, and geochemically unstable [1–4]. Conventional ground improvement techniques, including chemical grouting, deep mixing, and dynamic compaction, have been widely used [5–8]. However, these methods present several significant disadvantages, such as high energy consumption, substantial CO₂ emissions, and environmental risks, including groundwater contamination and ecosystem disruption. Additionally, these techniques often struggle to achieve uniform reinforcement in porous and cohesionless soils like calcareous sand due to irregular material penetration and uncontrollable diffusion paths [9–11].

In response to these limitations, Microbial-Induced Calcium Carbonate Precipitation (MICP) has emerged as a sustainable and biologically-driven alternative for soil stabilization. MICP utilizes ureolytic bacteria, such as *Sporosarcina pasteurii*, to catalyze the hydrolysis of urea, producing carbonate ions that, in the presence of calcium ions, precipitate as calcium carbonate (CaCO₃). This precipitation forms mineral bridges between soil particles, improving the soil’s strength, stiffness, and overall integrity. Compared to traditional methods, MICP has the advantage of being low-carbon, adaptable to in-situ conditions, and highly controllable through microbial activity, solution chemistry, and injection techniques [12–15].

Despite its potential, a major challenge in MICP implementation is achieving uniform reinforcement. In sandy soils, this nonuniformity often arises due to uneven microbial distribution, premature clogging, and variations in soil permeability. Calcareous sand, in particular, poses additional challenges because of its irregular, angular grains and high porosity. These characteristics contribute to a complex surface morphology and heterogeneous pore structure, which complicate the reinforcement process and reduce the overall effectiveness of MICP [16–19].

Previous studies have investigated factors influencing MICP efficiency, such as bacterial strain selection, nutrient media composition, and treatment duration [20–21]. However, less attention has been paid to the optimization of operational parameters such as infusion rate, cementation solution concentration, and the number of grouting cycles [22]. These parameters significantly impact both the spatial uniformity and the mechanical properties of the treated soil, and controlling them is essential for improving the consistency and overall effectiveness of the MICP process [23–25].

This study aims to fill this research gap by systematically investigating the effects of key operational parameters on the unconfined compressive strength (UCS) and reinforcement uniformity of MICP-treated calcareous sand [26]. A series of 75 UCS tests were conducted using *Sporosarcina pasteurii* and controlled one-dimensional column tests, where infusion rates, cementation concentrations, and grouting frequencies were varied. The coefficient of variation (C_v_) was used to quantify spatial reinforcement uniformity [27–28]. The results provide theoretical insights and practical recommendations for optimizing MICP treatment in calcareous sand, advancing its application in marine geotechnical engineering.

## 2. Materials and methods

### 2.1. Test soil samples

**Fig 1:**
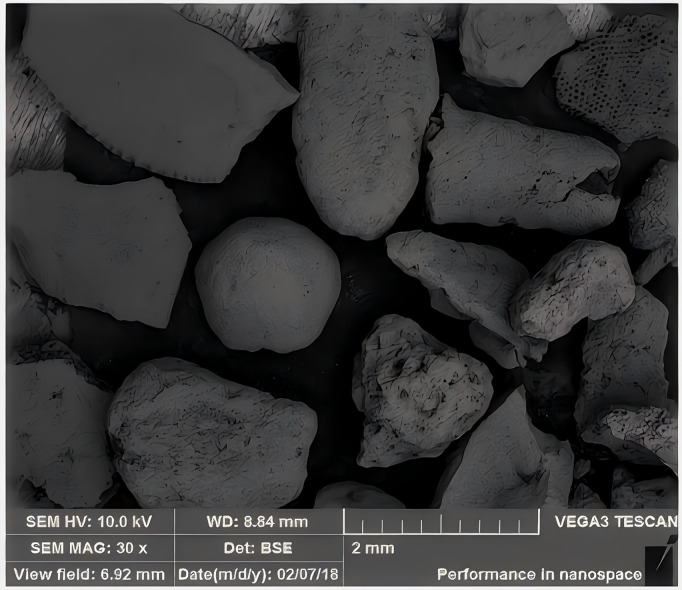
Calcareous sand and its scanning electron microscopy image.

The calcareous sand used in the experiments was collected from an island and reef in the South China Sea. Fig 1 shows a scanning electron microscopy (SEM) image of the sand particles. Unlike terrestrial sand, calcareous sand exhibits characteristics such as porous surfaces, irregular shapes, and angular edges, which result in significantly different mechanical behaviors compared to quartz sand.

In the model foundation preparation, a water sedimentation method was employed to form a saturated calcareous sand layer, ensuring uniform particle deposition and complete saturation. This approach minimizes segregation and simulates the natural depositional environment. After sedimentation, the sand layer was allowed to self-consolidate under its own weight, and a compacted clay layer was placed above to ensure a flat and stable overburden.

**Fig 2:**
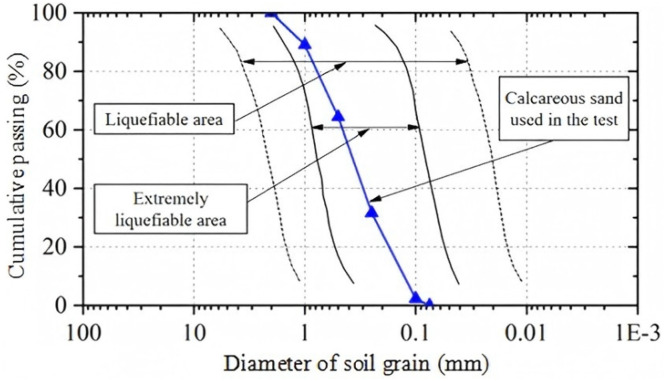
Grading curve of the calcareous sand used in the experiment.

The particle size distribution of the calcareous sand used in this study is shown in Fig 2. The coefficient of uniformity (C_u_) is 3.55, indicating a well-graded particle distribution. The coefficient of curvature (Cc) is 0.97, and the mean particle size d_50_ is 0.33 mm. Other physical properties include a particle density (G_s_) of 2.73 g/cm³, a maximum void ratio (e_Max_) of 1.44, and a minimum void ratio (e_Min_) of 1.02.

To quantify the density state, the relative density (Dr) of the saturated calcareous sand was estimated using the following formula:


$$D_{r}=\frac{e_{\max}-e}{e\max-e_{\min}}\times 100\%$$ (1)

Based on laboratory measurements and the known material properties, the void ratio of the saturated sand layer was estimated to be approximately 1.18, resulting in a calculated relative density of approximately 62%. This corresponds to a medium-dense condition, representative of naturally deposited marine sands and suitable for evaluating the mechanical response under MICP treatment.

### 2.2. Microbial cultivation

**Fig 3:**
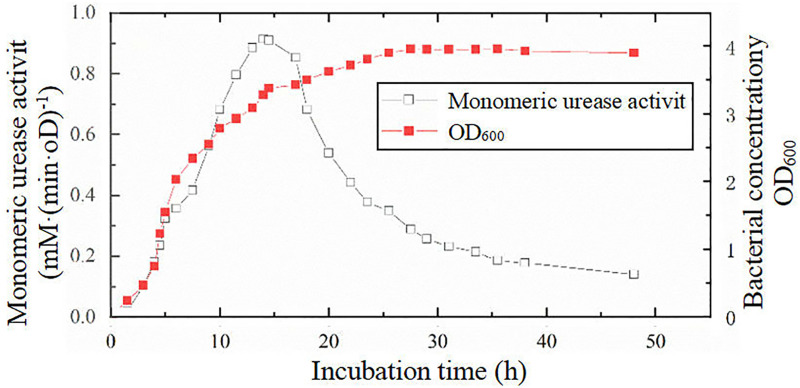
Changes in bacterial concentration and monomeric urease activity over time.

For the experiments, the Sporosarcina Pasteurii strain was selected, and the main components and contents of the culture medium were as follows: yeast extract, 20 g/L; NH4Cl, 10 g/L; MnCl2∙H2O, 12 mg/L; and NiCl2∙6 H2O, 24 mg/L. The solvent was distilled water, and the pH value of the culture medium was adjusted to 9.0 with a standard NaOH solution. After the prepared culture medium was sterilised by high-temperature steam at 121°C for 30 min, it was placed on an ultraclean workbench and cooled for later use. During the cooling process, an ultraviolet lamp was utilized to continue the sterilisation of the medium. The amount of bacteria used in the experiment was 10 L, so an industrial fermentation tank was used to expand the cultivation of the strains (30°C, 12–24 h of cultivation), with a strain inoculation ratio of 1:10. The variations in microbial monomer urease activity and concentration with culture incubation time are shown in Fig 3.

### 2.3. Microbial infusion reinforcement test on calcareous sand columns

**Fig 4:**
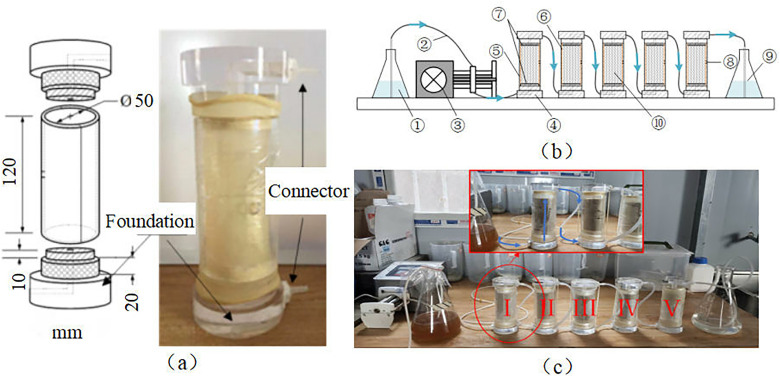
Schematic diagram of MICP reinforcement: (a) Mould; (b) Reinforcement diagram(1-Bacterial/gelling solution, 2-Peristaltic tube, 3-Peristaltic pump, 4-PVC base, 5-PVC Removable semicylindrical mould, 6-Rubber membrane, 7-Filter paper, 8-Pumping port, 9-Waste liquid, 10- Sand sample.); (c) Physical picture.

In this work, an experimental device was developed to test the reinforcement effect of soil samples at different distances from the grouting port, as shown in Fig 4. Five specially designed moulds were connected at both ends to form a sand column with a length of 50 cm and a diameter of 5 cm, which was used to simulate the soil at various positions. This method does not require cutting of the sand column, is easy to perform, and can accurately measure the strength of the soil at various distances from the port. By analysing the difference coefficient of the strength of the sand sample at different positions, the uniformity of the sample reinforcement can be obtained.

After the sand column model was completed, it was allowed to stand for 6 h to fully solidify the sand sample. A mixture of the bacterial mixture and low-concentration calcium chloride solution was injected into the model at a rate of 180 mL/h via a peristaltic pump, with 600 mL of cementing solution injected in each round. After the completion of cementing solution injection, the mixture was allowed to react for a period before proceeding to the next round of injection. The sum of the injection time and the settling time used for each round of cementing solution injection was the same, both of which were 24 h. After meeting the reinforcement requirements, 600 mL of distilled water was pumped into the sample with a peristaltic pump at a rate of 100 mL/h to remove residual bacterial and cementing fluids from the sample. After demoulding and sampling, the reinforced sample was immersed in deionised water for a period, and an unconfined compressive test was conducted on the MICP-reinforced calcareous sand.

### 2.4. Test conditions

The experiment considers the effects of the injection speed, concentration of bonding solution, and number of injection rounds on the uniformity of the reinforcement. Three infusion rates, namely, 0.5 mL/min, 3 mL/min, and 7 mL/min, were employed. The concentrations of the bonding fluid were considered 0.25 mol/L, 0.5 mol/L, 1 mol/L, and 3 mol/L, respectively. To investigate the influence of the number of injection wheels on the uniformity of reinforcement, four types of injection wheels were designed for the experiment, namely, 2, 4, 6, and 8 wheels, totalling 15 sets of experiments. Each set of experiments included 5 samples, and 75 unconfined compressive tests were conducted. A summary of the test conditions is shown in Table 1.

**Table 1:** One-dimensional sand column test conditions.

Working condition group	Working condition	Infusion rate (mL/min)	Cementing solution concentration (mol/L)	Infusion times
**M1**	M1-1	0.5	0.5	2
	M1-2	0.5	0.5	4
	M1-3	0.5	0.5	6
	M1-4	0.5	0.5	8
**M2**	M2-1	3	0.5	2
	M2-2	3	0.5	4
	M2-3	3	0.5	6
	M2-4	3	0.5	8
**M3**	M3-1	7	0.5	2
	M3-2	7	0.5	4
	M3-3	7	0.5	6
	M3-4	7	0.5	8
**M4**	M4-1	0.5	0.25	6
	M4-2	0.5	1	6
	M4-3	0.5	3	6

## 3. Experimental results and analysis

**Fig 5:**
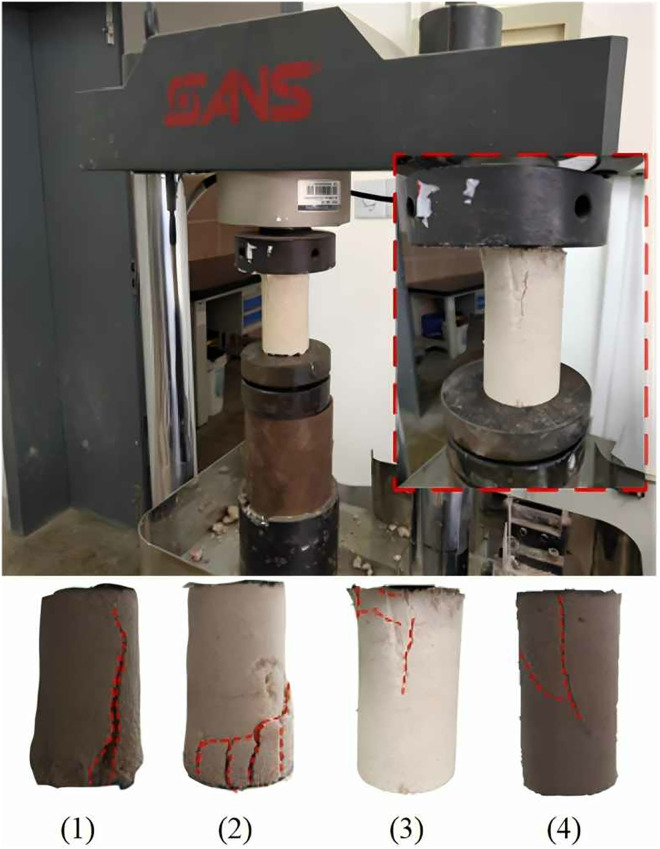
Reinforced sample destruction mode.

This experiment employed five sequentially connected 10 cm-long reinforcement chambers to obtain soil specimens at varying distances from the grouting port. Unconfined compressive strength (UCS) of these samples was measured to evaluate reinforcement effectiveness and uniformity within a 50 cm range. UCS was measured using standard unconfined compression tests on cylindrical samples after microbial treatment. UCS values were used to evaluate both strength development and reinforcement uniformity under different grouting conditions. As UCS serves as a reliable indicator of mechanical performance in microbially treated calcareous sand, its spatial variation under different reinforcement conditions was used to assess MICP-induced strength distribution. To quantitatively describe reinforcement uniformity, the coefficient of variation (C_v_) was introduced, enabling comparison of strength dispersion across different treatment scenarios and facilitating development of optimized microbial grouting protocols. Typical failure patterns observed in MICP-treated samples are illustrated in Fig 5. Failure modes varied significantly depending on cementation degree and spatial uniformity, classified into three categories:

Type I - Splitting failure (Fig 5(1)): Characterized by a vertically oriented, penetrating crack along the loading axis. Typically occurs in weakly reinforced specimens with insufficient interparticle bonding, where axial loading induces tensile stresses exceeding material tensile strength. No significant shear displacement is observed.

Type II - Localized failure (Fig 5(2)): In specimens with non-uniform cementation, weak zones develop internally. These fail progressively under loading, leading to localized fractures and multiple discontinuities, compromising structural coherence.

Type III - Shear failure (Fig 5(3) and (4)): In well-reinforced and uniformly treated samples, failure occurs along an inclined shear plane. This brittle failure mode features rapid post-peak stress drop, indicating strong bonding and high stiffness.

These observations confirm that MICP treatment enhances both strength and failure behavior of calcareous sand. Notably, failure mode is influenced not merely by peak strength, but also by reinforcement uniformity. Therefore, achieving both high strength and consistent performance in MICP-treated soils requires careful control of grouting parameters including flow rate, cementation concentration, and injection cycles.

### 3.1. Stress‒strain relationships of the cured sand samples

**Fig 6:**
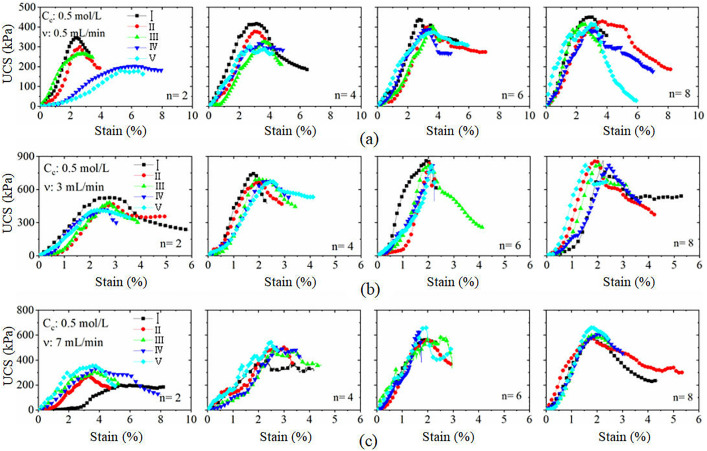
Variation of unconfined compressive strength under different (injection rates/ grouting rounds/ cementation concentrations): (a) Speed of 0.5 mL/min; (b) Speed of 3 mL/min;Speed of 7 mL/min.

To facilitate the analysis, the specimens were numbered according to Ⅰ, Ⅱ, Ⅲ, Ⅳ, and Ⅴ along the direction of filling; that is, specimen Ⅰ is the closest to the filling mouth, and specimen Ⅴ is the farthest from the filling mouth. Fig 6 shows the stress‒strain curves of the soil samples at different filling speeds and the number of filling rounds. As shown in the figure, according to the development trend of stress and strain, the stress‒strain curve can be roughly divided into three stages: slow growth of stress, rapid growth of stress and sudden drop of stress.

In Stage 1, the stress increases slowly due to the existence of pores between the sample particles. The load applied at this stage plays a role in the compression density of the sample, and the stress develops slowly with strain. In Stage 2, with continuous loading, the sample enters the stage of rapid growth of the stress; at this time, the load is mainly borne by the cementing structure of the soil. In Stage 3, when the applied load exceeds the compressive strength of the cementing structure of the sample, the sample is damaged, and the stress decreases abruptly. However, there is still a certain residual strength. In addition, the reinforcement of the harder specimens shows obvious strain softening, with typical brittle damage characteristics. In addition, the higher the degree of reinforcement is, the more obvious the brittle damage phenomenon.

Notably, the stress‒strain curves of some specimens have very irregular shapes (Fig 6c), the compaction stage is short. Moreover, when the stress is loaded to a certain value, the stress‒strain curves experience a sudden decrease and then a sudden increase in the stress, i.e., a phased stress peak. This has also been reported in unconfined tests of hydraulic soil. The main reason for the above phenomenon is that there is unevenness in the reinforcement of the specimen. Local cracks will appear inside the specimen, so the stress reveals a steep decrease. With further loading, the local cracks are compacted, and the internal stress of the specimen is redistributed, as the stress continues to increase.

**Fig 7:**
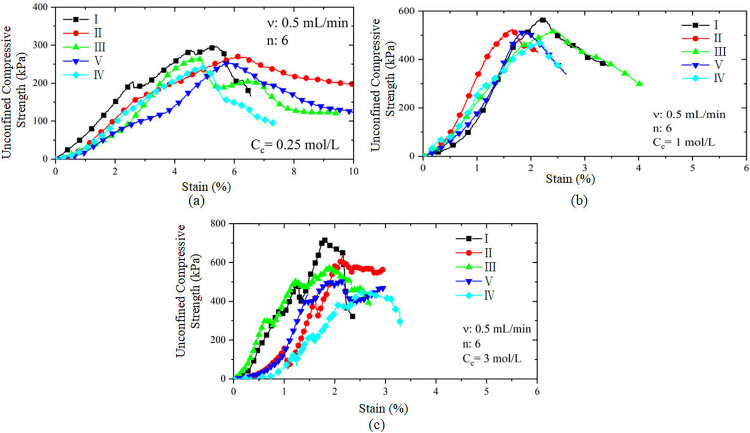
Stress-strain curves under unconfined compression for samples with different cementation concentrations: (a) Concentration of 0.25 mol/L; (b) Concentration of 1 mol/L; (c) Concentration of 3 mol/L.

Fig 7 shows the stress-strain curves under unconfined compression of the sand samples infused with six cementing solutions under different cementing solution concentration conditions. The trend of the stress–strain curves of each specimen is basically the same, and all of them clearly exhibit a strain softening phenomenon. A comparison of the compressive strength curves at the three different concentrations reveals that when the binder concentration Cc is 0.25 mol/L, the strain corresponding to the peak stress is 5~6%, which is much greater than the strain corresponding to a Cc of 3 mol/L. This indicates that the lower the binder concentration is, the greater the deformation capacity of the specimen, and the greater the strain required to reach the peak strength. In addition, combining the stress‒strain curves and the previous analyses, it can be seen that the reinforcement uniformity of Case M4-3 (Fig 7c) is relatively poor. Furthermore, the reinforcement uniformity of the specimens will be systematically analysed later, so it will not be repeated here.

### 3.2. Influence of the number and speed of cementing solution filling on the uniformity of the reinforcement

**Fig 8:**
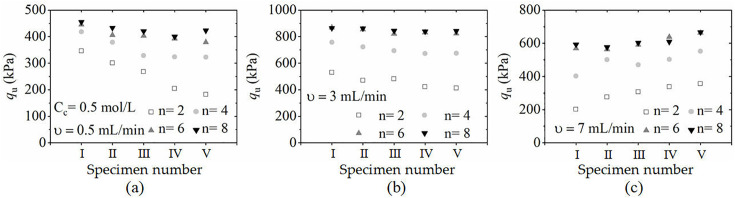
Distribution of the unconfined compressive strength of specimens at different positions under different filling speeds: (a) Working condition M1; (b) Working condition M2; (c) Working condition M3.

To compare the distributions of the peak stress q_u_ (compressive strength) of the specimens under different reinforcement conditions more clearly, the peak strength of each specimen at different locations along the grouting opening is shown in Fig 8. As shown in the figure, the strength of the specimens increased significantly in the first 4 rounds of grouting reinforcement; for example, in Case M1, the strength of the sand column closest to the grouting mouth was approximately 425 kPa after 4 rounds of grouting, which was approximately 20% greater than that in the first 2 rounds of grouting. The strength of the specimens at each location basically did not increase after 6 rounds of grouting reinforcement. This finding indicated that the bacteria had essentially lost urease activity after 6 rounds of grouting reinforcement. The stagnation in strength beyond 6 rounds is attributed to diminished bacterial activity in the stored solution and precipitation site saturation, not intrinsic limitations of fresh bacterial batches.

A comparison of M1, M2 and M3 reveals that the filling speed of the cementing liquid not only has a significant effect on the reinforcement strength but also affects the soil strength distribution along the filling direction.

When the filling speed is 0.5 mL/min, the greater the distance from the filling port is, the lower the soil cementation strength, which is due to two main reasons. First, the mineralisation of microorganisms leads to a continuous loss of cementation liquid in the process of transport. Therefore, the permeability coefficient of the soil body at a position far from the cementation port reduces the reinforcing effect of the soil body at this location.

When the filling speed of the cementing liquid is increased to 7 mL/min, the soil strength distribution changes. Particularly, the closer the soil is to the filling mouth, the lower the soil cementing strength is, and the soil at the location of the grout outlet is relatively high. The main reason for this phenomenon is that the higher filling rate removes the microorganisms adsorbed on the surface of the calcareous sand particles and generates calcium carbonate crystals, resulting in a relatively poor cementation effect at the filling outlet. Thus, selecting the appropriate cementing solution infusion rate can improve the cementing efficiency and reinforcement uniformity.

Reinforcement strength and uniformity are two important indices for evaluating the reinforcing effect of MICP, so the two parameters of unconfined compressive strength qu and coefficient of variation C_v_ were used to evaluate the reinforcing strength and uniformity of MICP. C_v_ refers to the percentage of standard deviation from the mean of a set of data and is mainly used to describe the degree of dispersion of the data. The larger the coefficient of variation is, the more inhomogeneous the reinforcement is. C_v_ can be calculated via the following formula:


$$C_{v}=\frac{S}{M}\times 100\%$$ (2)

Here, $S=\sqrt{\sum_{i=1}^{n}\frac{{\left(q_{ui}-M\right)}^{2}}{n}}$ is the standard deviation; $M=\frac{\sum_{i=1}^{n}q_{ui}}{n}$ is the mean value and qui is the qu of specimen number i, n = 5.

**Fig 9:**
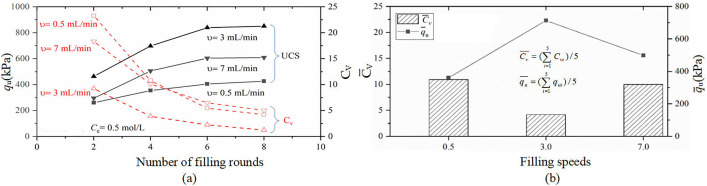
Effect of the cement filling speed on unconfined compressive strength q_u_ and uniformity (C_v_): (a) Changes in qu and C_v_ with increasing number of instillations at different instillation speeds(0.5, 3, and 7 mL/min); (b) The variation in qu and C_v_ with the filling speed of the cementing liquid (mL/min).

Fig 9 compares the unconfined compressive strength (q_u_) and the coefficient of variation (C_v_) of MICP-treated specimens under different injection rates of the cementation solution (0.5, 3, and 7 mL/min). As shown in Fig 9a, with an increasing number of filling rounds, q_u_ increases progressively and eventually stabilizes, while C_v_ decreases accordingly. This trend indicates that repeated grouting significantly enhances both the compressive strength and spatial uniformity of reinforcement. For instance, at an injection rate of 3 mL/min, the average q_u_ rises from approximately 500 kPa to 800 kPa after eight rounds, while C_v_ drops to around 1%, only 12% of its initial value after the second round.

Fig 9b further illustrates the influence of injection rate. Both q_u_ and C_v_ exhibit a non-monotonic trend, with optimal results achieved at 3 mL/min. This rate yields the highest strength and lowest variability, suggesting an optimal balance between bacterial activity and cementation uniformity. At the lower rate of 0.5 mL/min, the cementation solution tends to precipitate prematurely near the inlet due to slower flow and high microbial concentration, limiting its penetration and leading to uneven reinforcement. In contrast, at the higher rate of 7 mL/min, the rapid flow may wash out bacteria before adequate CaCO₃ precipitation occurs, resulting in weak bonding and greater strength variability.

These findings demonstrate that a moderate injection rate (3 mL/min) ensures sufficient bacterial transport and reaction time, promoting both higher strength and improved uniformity. Therefore, precise control of injection rate is critical for optimizing MICP performance in calcareous sand.

It is also noted that after six filling rounds, the increase in strength begins to plateau. This stagnation is not necessarily attributed to reduced urease activity—since fresh bacterial solutions were used in each round—but rather to accumulated physical and chemical effects within the sand matrix. These include local clogging of pores by CaCO₃, reduced permeability, and limited transport of nutrients or oxygen, all of which impede further in-situ precipitation and reduce the efficiency of subsequent MICP reactions.

### 3.3. Effect of binder concentration on reinforcement uniformity

**Fig 10:**
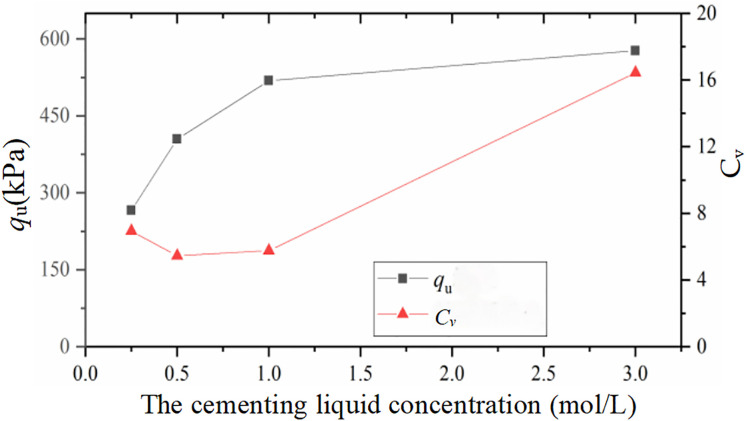
Influence of the cementing liquid concentration on the reinforcement effect.

The stress‒strain curves of the specimens at different binder concentrations in Section 2.1 indicate that the binder concentration strongly influences the reinforcement effect. The mean values of the soil compressive strength and its coefficient of variation under different binder concentrations are given in Fig 10. Fig10 shows that when the binder concentration was increased from 0.25 mol/L to 0.5 mol/L, the mean value of the unconfined compressive strength (q_u_) of the soil samples increased from approximately 260 kPa to 400 kPa, which is a large increase of approximately 53%. With increasing cementing liquid concentration, the increase in compressive strength decreases continuously because a cementing liquid concentration that is too high greatly reduces microbial urease activity, which inhibits the calcium carbonate crystallisation process.

A comparison of the coefficients of variation of the compressive strength of the soil samples with different binder concentrations reveals that the coefficient of variation of the compressive strength C_v_ of the soil samples with a binder concentration of 3 mol/L is relatively large, approximately 16%, which is 2.6 times greater than that of the reinforced samples with a binder concentration of 0.5 mol/L. This indicates that a higher binder concentration leads to obvious unevenness in the reinforcement and that lowering the concentration of the binder can effectively improve the uniformity of the reinforcement.

In summary, a binder concentration that is too low will lead to low cementation efficiency, whereas a binder concentration that is too high will cause significant consolidation nonuniformity.

In the practical application of the MICP method to treat calcareous sand foundations, the appropriate binder concentration should be selected, accounting for both the binder efficiency and reinforcement uniformity. To obtain a more satisfactory reinforcing effect, the binder concentrations used in the subsequent experiments of this paper were 0.5 mol/L and 1 mol/L.

To quantitatively assess the variability in the mechanical response of specimens under different treatment conditions, the coefficient of variation (C_v_) was used as a measure of strength dispersion across each group. This parameter reflects the degree of uniformity and consistency in the unconfined compressive strength (UCS) values at different positions along the sand column. As shown in Figs 8–10, the C_v_ values varied significantly with changes in filling rate, cementation concentration, and the number of filling rounds. Lower C_v_ values indicate more homogeneous reinforcement, while higher values suggest non-uniform MICP effects. This statistical representation of variability strengthens the validity of comparing different experimental conditions and provides a more reliable basis for optimization.

### 3.4. Relationships between the unconfined compressive strength q_u_ and E_50_ or ε_f_

**Fig 11:**
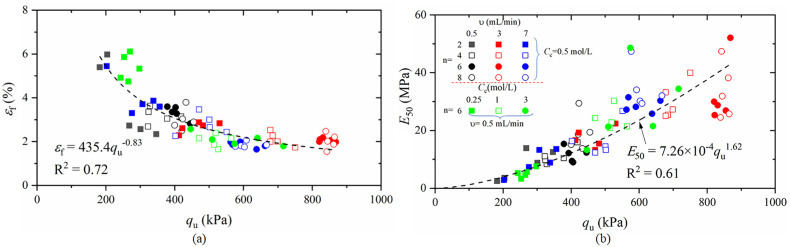
Relationships between the unconfined compressive strength and destructive strains εf and E_50_: (a) Relationship between the destructive strain εf and peak strength q_u_; (b) Relationship between the modulus of elasticity E50 and peak strength q_u._


The stress‒strain relationship of the soil obtained from the unconfined compressive test mainly depends on three parameters, i.e., the compressive strength q_u_ (peak stress), the destructive strain ε_f_ corresponding to the peak stress q_u_, and the modulus of elasticity (E_50_), of which E_50_ is an important parameter required for deformation analysis. Here, E_50_ is defined as the ratio of the stress to the strain that corresponds to the stress value when the stress value reaches 50% of the peak stress for the first time. The relationships among the destructive strain ε_f_, modulus of elasticity E_50_ and peak strength q_u_ of the specimen are given in Fig 11. As shown in Fig 11a, the destructive strain εf of the cured specimen has an inverse correlation with the peak strength qu, and ε_f_ is distributed between 1.5% and 6%. ε_f_ and q_u_ can be regressed with a power function, and the specific fitting equations are as follows:

$$\varepsilon_{f}=435.4q_{u}^{-0.83}$$ (3)

The coefficient of determination R_2_ is 0.72, which is a good fit. This indicates that it is appropriate to use the power function to reflect the relationship between ε_f_ and q_u_.

As shown in Fig 11b, the modulus of elasticity E_50_ of the reinforced calcareous sand samples has a positive correlation with the peak strength q_u_, i.e., the higher the compressive strength of the reinforced samples is, the greater the modulus of elasticity. Like εf and qu, E_50_ and qu can also be fitted by a power function, and the fitting equation is as follows:


$$E_{50}=7.26\times 10^{-4}q_{u}^{1.62}$$ (4)

The fit coefficient of determination R_2_ was 0.61.

In summary, in the study of one-dimensional (1D) microbial grouting for soil reinforcement, the inhomogeneity of 1D specimen reinforcement is often due to the uneven distribution of microorganisms within the specimen with uneven crystal-forming reactions. The strength of the soil at the grouting opening is high and decreases gradually with increasing distance from the opening. The selection of an appropriate grouting rate and binder concentration can improve the reinforcement efficiency and reduce the reinforcement nonuniformity.

## 4. Conclusions

This study developed a one-dimensional microbial grouting test system to investigate reinforcement performance and uniformity of calcareous sand treated using microbial-induced calcium carbonate precipitation (MICP). The effects of key injection parameters—including injection rate, cementation solution concentration, and injection cycles—on mechanical properties and uniformity of reinforced soil were systematically evaluated. The main conclusions are as follows:

(1) Mechanical enhancement and failure characteristics: MICP treatment significantly improved mechanical strength of calcareous sand. Stress-strain behavior of reinforced samples exhibited three-stage response: initial compaction, rapid strength increase, and brittle failure. Enhanced reinforcement led to greater stiffness and more pronounced brittle behavior. Non-uniform treatment resulted in irregular stress-strain curves and multiple peak stages, indicating localized failure zones.
(2) Influence of injection rate: Injection rate strongly affected both reinforcement strength and uniformity. A moderate injection rate of 3 mL/min provided optimal balance, producing highest unconfined compressive strength (UCS) and lowest coefficient of variation (C_v_). Lower rates caused premature CaCO₃ precipitation near the inlet, while higher rates risked bacterial washout and uneven cementation.
(3) Effect of cementation solution concentration: Binder concentration strongly affected MICP performance. Concentrations between 0.5 mol/L and 1 mol/L achieved both effective cementation and high uniformity. Low concentrations (<0.5 mol/L) resulted in insufficient CaCO₃ precipitation, while high concentrations (3 mol/L) reduced microbial activity and caused non-uniform reinforcement.
(4) Role of injection frequency: Increasing grouting cycles significantly enhanced both strength and uniformity. Strength improvement plateaued after six cycles, likely due to pore clogging and reduced permeability rather than microbial inactivity.
(5) Correlations among mechanical parameters: Failure strain (εf) decreased with increasing UCS, while elastic modulus (E50) increased. Both relationships followed power-law trends, enabling predictive modeling of MICP-treated soil behavior.
(6) Recommended parameters for field application: For optimal MICP performance in calcareous sand, an injection rate of 3 mL/min, binder concentration of 0.5–1 mol/L, and 6–8 grouting cycles are recommended. These parameters ensure effective, uniform reinforcement, supporting MICP application as a sustainable alternative to conventional soil stabilization techniques.

Microbial-Induced Calcium Carbonate Precipitation (MICP) presents a promising alternative for soil stabilization in geotechnical engineering, particularly in coastal and marine environments. Its applications include reinforcing weak soils, improving soil permeability, and providing a sustainable, eco-friendly method for foundation stabilization. However, MICP does have limitations, such as variability in treatment uniformity, potential microbial activity loss over time, and challenges related to controlling the grouting process, especially in large-scale projects. The effectiveness of MICP heavily depends on factors like injection speed, cementation concentration, and microbial strain choice. Engineers should consider these factors when applying MICP to ensure optimal results in both performance and cost-effectiveness.

## References

[1] El-Kelesh AM, Matsui T. Calibration Chamber Modeling of Compaction Grouting. Geotechnical Testing Journal. 2008;31(4):295–307. doi: 10.1520/gtj100792

[2] Liu X, Chen H, Liu Q, Liu B, He J. Modelling slurry flowing and analyzing grouting efficiency under hydro-mechanical coupling using numerical manifold method. Engineering Analysis with Boundary Elements. 2022;134:66–78. doi: 10.1016/j.enganabound.2021.09.030

[3] Akbulut S, Saglamer A. Estimating the groutability of granular soils: a new approach. Tunnelling and Underground Space Technology. 2002;17(4):371–80. doi: 10.1016/s0886-7798(02)00040-8

[4] Farhadian H, Maleki Z. Groutability classification of granular soils with cement grouts. Journal of Rock Mechanics and Geotechnical Engineering. 2023;15(6):1580–90. doi: 10.1016/j.jrmge.2022.09.007

[5] Karandi PK, Aishwarya T, Marbaniang C, Juneja A. Effect of biocementation on sand sample strength measured using direct shear tests. Proceedings of the Institution of Civil Engineers - Ground Improvement. 2024;177(5):342–56. doi: 10.1680/jgrim.23.00032

[6] Park S, Park J, Chang I. Laboratory assessment of shear strength parameters of sand amended via subsequent biopolymer-based soil treatment and enzyme-induced calcite precipitation combinations. IOP Conf Ser: Earth Environ Sci. 2024;1337(1):012038. doi: 10.1088/1755-1315/1337/1/012038

[7] Tang C-S, Li H, Pan X-H, Yin L-Y, Cheng L, Cheng Q, et al. Coupling effect of biocementation-fiber reinforcement on mechanical behavior of calcareous sand for ocean engineering. Bull Eng Geol Environ. 2022;81(4). doi: 10.1007/s10064-022-02662-7

[8] Xiao P, Liu H, Xiao Y, Stuedlein AW, Evans TM. Liquefaction resistance of bio-cemented calcareous sand. Soil Dynamics and Earthquake Engineering. 2018;107:9–19. doi: 10.1016/j.soildyn.2018.01.008

[9] Tekin E, Akbas SO. Predicting groutability of granular soils using adaptive neuro-fuzzy inference system. Neural Comput & Applic. 2017;31(4):1091–101. doi: 10.1007/s00521-017-3140-3

[10] Mujah D, Cheng L, Shahin MA. Microstructural and Geomechanical Study on Biocemented Sand for Optimization of MICP Process. J Mater Civ Eng. 2019;31(4). doi: 10.1061/(asce)mt.1943-5533.0002660

[11] Zhou X-Z, Chen Y-M, Li W-W, Liu H-L. Monotonic and cyclic behaviors of loose anisotropically consolidated calcareous sand in torsional shear tests. Marine Georesources & Geotechnology. 2018;37(4):438–51. doi: 10.1080/1064119x.2018.1449274

[12] Wang J, Alidekyi SN, Nong X, Huang J, Wang X. Thermal–hydraulic-mechanical-chemical-biological (THMCB) coupling in microbial induced carbonate precipitation (MICP): A comprehensive review. Environ Earth Sci. 2025;84(12). doi: 10.1007/s12665-025-12327-9

[13] Mehrabi R, Atefi-Monfared K. A coupled bio-chemo-hydro-mechanical model for bio-cementation in porous media. Can Geotech J. 2022;59(7):1266–80. doi: 10.1139/cgj-2021-0396

[14] Cheng L, Shahin MA, Cord-Ruwisch R. Bio-cementation of sandy soil using microbially induced carbonate precipitation for marine environments. Géotechnique. 2014;64(12):1010–3. doi: 10.1680/geot.14.t.025

[15] Cheng L, Shahin MA. Urease active bioslurry: a novel soil improvement approach based on microbially induced carbonate precipitation. Can Geotech J. 2016;53(9):1376–85. doi: 10.1139/cgj-2015-0635

[16] Yuan J, Lei D, Shan Y, Tong H, Fang X, Zhao J. Direct Shear Creep Characteristics of Sand Treated with Microbial-Induced Calcite Precipitation. Int J Civ Eng. 2022;20(7):763–77. doi: 10.1007/s40999-021-00696-8

[17] Cardoso R, Pires I, Duarte SOD, Monteiro GA. Effects of clay’s chemical interactions on biocementation. Applied Clay Science. 2018;156:96–103. doi: 10.1016/j.clay.2018.01.035

[18] Ugur GE, Rux K, Boone JC, Seaman R, Avci R, Gerlach R, et al. Biotrapping Ureolytic Bacteria on Sand to Improve the Efficiency of Biocementation. ACS Appl Mater Interfaces. 2024;16(2):2075–85. doi: 10.1021/acsami.3c13971 38176018

[19] Hanisch P, Pechtl M, Maurer H, Maier F, Bischoff S, Nagy B, et al. The effect of different additives on bacteria adsorption, compressive strength and ammonia removal for MICP. Environ Earth Sci. 2024;83(22). doi: 10.1007/s12665-024-11929-z

[20] Lapierre FM, Schmid J, Ederer B, Ihling N, Büchs J, Huber R. Revealing nutritional requirements of MICP-relevant Sporosarcina pasteurii DSM33 for growth improvement in chemically defined and complex media. Sci Rep. 2020;10(1):22448. doi: 10.1038/s41598-020-79904-9 33384450 PMC7775470

[21] Kahani M, Kalantary F, Soudi MR, Pakdel L, Aghaalizadeh S. Optimization of cost effective culture medium for Sporosarcina pasteurii as biocementing agent using response surface methodology: Up cycling dairy waste and seawater. Journal of Cleaner Production. 2020;253:120022. doi: 10.1016/j.jclepro.2020.120022

[22] Liu Y, Hu K, Pan M, Dong W, Wang X, Zhu X. Research and Application of Green Technology Based on Microbially Induced Carbonate Precipitation (MICP) in Mining: A Review. Sustainability. 2025;17(17):7587. doi: 10.3390/su17177587

[23] Chen Y, Zhang R, Zi J, Han J, Liu K. Evaluation of the treatment variables on the shear strength of loess treated by microbial induced carbonate precipitation. J Mt Sci. 2025;22(3):1075–86. doi: 10.1007/s11629-024-9100-3

[24] Wang Y, Konstantinou C. A Comprehensive Optimization Study of Microbially Induced Carbonate Precipitation for Soil Strength Enhancement: Impact of Biochemical and Environmental Factors. J Geotech Geoenviron Eng. 2024;150(10). doi: 10.1061/jggefk.gteng-12230

[25] Baziar MH, Alibolandi M. Liquefaction Evaluation of Microbial Induced Calcium Carbonate Precipitation (MICP) Treated Sands; A Strain Energy Approach. Journal of Earthquake Engineering. 2023;27(15):4512–25. doi: 10.1080/13632469.2023.2171508

[26] Omoregie AI, Palombo EA, Ong DEL, Nissom PM. Biocementation of sand by Sporosarcina pasteurii strain and technical-grade cementation reagents through surface percolation treatment method. Construction and Building Materials. 2019;228:116828. doi: 10.1016/j.conbuildmat.2019.116828

[27] Bernardi D, DeJong JT, Montoya BM, Martinez BC. Bio-bricks: Biologically cemented sandstone bricks. Construction and Building Materials. 2014;55:462–9. doi: 10.1016/j.conbuildmat.2014.01.019

[28] Yasuhara H, Neupane D, Hayashi K, Okamura M. Experiments and predictions of physical properties of sand cemented by enzymatically-induced carbonate precipitation. Soils and Foundations. 2012;52(3):539–49. doi: 10.1016/j.sandf.2012.05.011

